# Quantitative Analysis of Neurotransmitter Pathways Under Steady State Conditions – A Perspective

**DOI:** 10.3389/fendo.2013.00179

**Published:** 2013-11-18

**Authors:** Arthur J. L. Cooper

**Affiliations:** ^1^Department of Biochemistry and Molecular Biology, New York Medical College, Valhalla, NY, USA

**Keywords:** branched-chain amino acid branched-chain keto acid shuttle, GABA–glutamine cycle, glutamate–glutamine cycle, metabolic compartmentation, cerebral nitrogen metabolism

## Abstract

In a contribution to this Research Topic Erkki Somersalo and Daniela Calvetti carried out a mathematical analysis of neurotransmitter pathways in brain, modeling compartmental nitrogen flux among several major participants – ammonia, glutamine, glutamate, GABA, and selected amino acids. This analysis is important because cerebral nitrogen metabolism is perturbed in many diseases, including liver disease and inborn errors of the urea cycle. These diseases result in an elevation of blood ammonia, which is neurotoxic. Here, a brief description is provided of the discovery of cerebral metabolic compartmentation of nitrogen metabolism – a key feature of cerebral glutamate–glutamine and GABA–glutamine cycles. The work of Somersalo and Calvetti is discussed as a model for future studies of normal and pathological cerebral ammonia metabolism.

The concept of compartmentation of cerebral nitrogen metabolism originated with the classic work of Berl et al. (1). These authors infused large (non-tracer) doses of [^15^N]ammonia into cats and determined the relative enrichment of ^15^N in cerebral glutamate, glutamine (amine), and glutamine (amide). Glutamine synthetase (GS) catalyzes the conversion of ammonia and glutamate to glutamine (Eq. 1). A portion of this glutamate was labeled with ^15^N as a result of the action of glutamate dehydrogenase (GDH) (Eq. 2). Thus, if the brain were acting as a single metabolic compartment, the relative enrichment of label should have been greater in glutamate precursor than in glutamine (amine). However, the relative enrichment of ^15^N was found to be: glutamine (amide) > glutamine (amine) > glutamate. This finding prompted Berl et al. (1) to suggest that in the brain, glutamate is rapidly turned over to glutamine in a small compartment that is distinct from a larger compartment of more slowly turning over glutamate. We later verified cerebral compartmentation using intracarotid and intraventricular infusion of *tracer* quantities of [^13^N]ammonia into rats (2). Importantly, Norenberg and colleagues showed that in brain parenchyma GS is present exclusively in astrocytes (3, 4). [See also the article in this Research Topic by Derouiche and Anlauf.] Thus, ammonia entering the brain largely by diffusion of ammonia free base (NH_3_) (2, 5, 6) is rapidly converted to glutamine (amide) in the small compartment (i.e., astrocytes). The large compartment approximately represents the neurons, which contain a portion of the GDH activity of the brain.

(1) $$\text{L-Glutamate}+NH_{3}+\mathrm{ATP}⇄\text{L-glutamine}+\mathrm{ADP}+P_{i}$$

(2) $$\begin{array}{llll} & \alpha -\mathrm{Ketoglutarate}+{\mathrm{NH}}_{4}^{+}+\mathrm{NAD(P)H}⇄\text{L-glutamate}\; & & \;\\ & \;+\mathrm{NAD}{\left(P\right)}^{+}+H_{2}O \end{array}$$

Compartmentation plays a pivotal role in the cerebral glutamate–glutamine cycle. Thus, glutamate released during neurotransmission is taken up in the small compartment (astrocytes), where it is converted to glutamine by GS. Glutamine is released from the astrocytes to the large compartment (neurons) where it is hydrolyzed by glutaminase (phosphate-activated glutaminase; PAG; Eq. 3) to glutamate and ammonia, completing the cycle (7, 8).

(3) $$\text{L-Glutamine}+H_{2}O\to \text{L-glutamate}+NH_{3}$$

The glutamate–glutamine cycle is not strictly stoichiometric and is “leaky.” For example, net uptake of many amino acids occurs across the human blood–brain barrier (BBB) (9). The nitrogen obtained from this source contributes to the astrocytic pool of nitrogen in glutamate and glutamine. Moreover, not all astrocytic glutamine is released to the neurons. To maintain nitrogen balance cerebral glutamine is released to the extracellular fluids and circulation (10). Nevertheless, the concept of a glutamate–glutamine cycle has been useful in furthering our understanding of nitrogen homeostasis in the brain. The flux through this cycle is extremely rapid, estimated to be about 80% the rate of glucose oxidation (11).

[^13^N]Ammonia has proved useful in positron-emission tomography (PET) studies of cerebral ammonia metabolism in controls, cirrhotics with non-overt encephalopathy, and cirrhotics with overt encephalopathy (12, 13). The main finding is that cerebral ammonia metabolism is enhanced due to increased blood ammonia in cirrhotic patients, but the kinetics of cerebral ammonia uptake and metabolism are not affected by hyperammonemia. Thus, owing to the higher concentration of ammonia in the blood, more ammonia will enter the brain in hyperammonemic patients than in normoammonemic individuals, despite the fact that tracer uptake by the brain is similar in the two groups. Because metabolic trapping of [^13^N]ammonia is similar in both cases, the findings suggest that the concentration of ammonia in astrocytes is below the *K*_m_ value exhibited by GS toward ammonia even in the hyperammonemic individuals. In this regard, the whole brain ammonia concentration in the rat is estimated to be ∼180 μM (10), a value identical to the *K*_m_ for ammonia exhibited by ovine brain GS (14). Moreover, as discussed below, the ammonia concentration in astrocytes is likely to be even lower than that of intact brain. Some evidence suggests that the specific activity of cerebral GS is decreased in portacaval-shunted rats (a model of chronic liver disease) (15, 16). Nevertheless, increased influx of ammonia in hyperammonemic individuals will result in increased synthesis of astrocytic glutamine. Owing to the uncertainty of the specific activity of [^13^N]ammonia in the astrocytes it is not yet possible to assign an absolute value for glutamine synthesis in the brains of normal and cirrhotic patients using PET. Nevertheless, the trapping of blood-derived [^13^N]ammonia in human brain is consistent with a rapid nitrogen flow through the glutamate–glutamine cycle.

A GABA–glutamine cycle also operates in the brain. Inhibitory neurotransmitter GABA released by neurons is taken up in part by astrocytes. In the astrocytes, GABA is transaminated to succinic semialdehyde, which is oxidized to succinate by succinic semialdehyde dehydrogenase, allowing the 4-carbon unit of GABA to enter the tricarboxylic acid (TCA) cycle (the GABA shunt). Glutamine is then released to the neurons and converted to glutamate. A portion of this glutamate is decarboxylated to GABA, thereby completing the GABA–glutamine cycle (17, 18). In the brain, acetate is metabolized exclusively in astrocytes (19). Studies with [^13^C]acetate suggest that flux through the GABA–glutamine cycle in the neocortex of vigabatrin-treated rats is about 6% the rate through the TCA cycle (20). In a more recent study, van Eijsden et al. (21) calculated the flux through the glutamate–glutamine cycle, GABA–glutamine cycle, and GABA shunt in rat brain to be 0.274 ± 0.023, 0.033 ± 0.005, and 0.025 ± 0.006 μmol/min/g, respectively.

Most of the ammonia destined for glutamine (amide) synthesis in astrocytes is likely derived from ammonia taken up from the blood/cerebrospinal fluid (CSF) and from ammonia-generating reactions in both astrocytes and neurons. But how is the astrocytic glutamate (amine) pool maintained? In the simplest explanation, glutamate released from the neurons is stoichiometrically converted to glutamine in the astrocytes, but this is unlikely. Despite the fact that the GDH reaction is reversible (accounting for labeling of brain glutamate in tracer studies with [^13^N]ammonia/[^15^N]ammonia) it is likely that the *net* direction of the GDH reaction in astrocytes (22) and in whole brain is in the direction of glutamate oxidation (23). Thus, α-ketoglutarate generated by the GDH reaction in astrocytes must be converted back to glutamate to maintain glutamate balance (24, 25). This conversion can be accomplished by transamination of α-ketoglutarate. However, in order to maintain nitrogen homeostasis the amino acid transamination partner must be imported into astrocytes from the neurons or from the blood/CSF. As discussed below, various neuron-to-astrocyte amino acid shuttles have been proposed.

Waagepetersen and colleagues (26–29) suggested that ammonia is incorporated into glutamate in neurons via the GDH reaction (forward direction of Eq. 2) and thence into alanine by means of the alanine aminotransferase (ALAT)-catalyzed reaction (Eq. 4). Alanine is then transported into the astrocytes and transaminated with α-ketoglutarate to regenerate glutamate nitrogen (Eq. 5). In support of this notion both glutamine and alanine are greatly elevated in cerebral extracellular fluid in hyperammonemic patients with fulminant hepatic failure (30) and in a rat model of this disease (31). Carbon balance may possibly be maintained by transfer of lactate from astrocytes to neurons (the lactate–alanine cycle). Redox balance is maintained because GDH is proposed to catalyze net reductive amination of α-ketoglutarate in neurons (Eq. 2, forward reaction) and net oxidation of glutamate in astrocytes (Eq. 2; back reaction). However, tracer studies with ^15^N-labeled alanine, ammonia, and glutamine revealed no direct coupling of the glutamate–glutamine and lactate–alanine shuttles in cerebellar co-cultures (27). Moreover, the existence of an astrocyte-to-neuron lactate shuttle is controversial (32). Therefore, the following discussion will focus only on the part of the pathway suggested to transfer alanine nitrogen from neurons to astrocytes, which is referred to as the alanine shuttle.

Previous work has shown that (1) the specific activity of cerebral ALAT is relatively low (33), (2) even after a 20-min intracarotid infusion of [^13^N]ammonia into the rat brain, label cannot be detected in alanine despite labeling of glutamate (23), and (3) even after brain GS is inhibited 85% by the GS inhibitor l-methionine-*S*,*R*-sulfoximine (MSO) and the rats are hyperammonemic, relatively little label derived from intracarotid [^13^N]ammonia is incorporated into brain glutamate (2, 23). Thus, under normoammonemic conditions an alanine shuttle [i.e., ammonia → glutamate (neurons) → alanine (neurons) → alanine (astrocytes) → glutamate (astrocytes)] is likely to be of minor importance for replenishing glutamate nitrogen in astrocytes (23). Bak et al. (27), however, suggest that the alanine shuttle may be prominent under hyperammonemic conditions.

(4) $$\begin{array}{llll} & \mathrm{Pyruvate}+\text{L-glutamate}\to \text{L-alanine}\; & & \;\\ & \;+\alpha -\text{ketoglutarate (neurons)} \end{array}$$

(5) $$\begin{array}{llll} & \text{L-Alanine}+\alpha -\mathrm{ketoglutarate}\to \mathrm{pyruvate}\; & & \;\\ & \;+\text{L-glutamate (astrocytes)} \end{array}$$

Another nitrogen shuttle is the branched-chain amino acid/branched-chain α-keto acid (BCAA/BCKA) shuttle, first proposed by Hutson et al. (34, 35). These authors pointed out the unique distribution of the branched-chain aminotransferase (BCAT) isozymes in brain. The cytosolic isozyme (BCATc) is widely distributed in tissues, including brain, whereas the mitochondrial isozyme (BCATm) is confined to brain, placenta, and ovaries (34). In the brain, BCATc is more prevalent in neurons, whereas BCATm is more prevalent in the astrocytes (34). The presence of both BCAT isozymes in the brain suggests that they play an important role in maintaining cerebral BCAA homeostasis (34, 35). In the BCAA/BCKA shuttle, BCATc catalyzes the transamination of a BCKA, such as α-ketoisocaproate (KIC, the α-keto acid analog of leucine) with glutamate in neurons (Eq. 6). The resulting BCAA is taken up by astrocytes, where BCATm catalyzes the transfer of nitrogen from the BCAA to α-ketoglutarate, regenerating glutamate (Eq. 7). The KIC generated in the astrocytes is returned to the neurons conserving carbon balance.

(6) $$\begin{array}{llll} & \text{L-Glutamate}+\alpha -\mathrm{ketoisocaproate}\to \alpha -\mathrm{ketoglutarate}\; & & \;\\ & \;+\text{L-leucine (neurons)} \end{array}$$

(7) $$\begin{array}{llll} & \text{L-Leucine}+\alpha -\mathrm{ketoglutarate}\to \alpha -\mathrm{ketoisocaproate}\; & & \;\\ & \;+\text{L-glutamate (astrocytes)}\; \end{array}$$

The notion that astrocytes metabolize leucine has strong support. Leucine is taken up across the human BBB (9) and has a relatively high brain uptake index in rats (36). Moreover, Berl and Frigyesi (37, 38) previously showed that leucine is metabolized in the small compartment in cat brain. As pointed out by Yudkoff et al. (39, 40), transfer of leucine nitrogen to α-ketoglutarate is favorable in brain, especially in astrocytes. Isoleucine, another BCAA, is of interest because its metabolism will not only replenish glutamate nitrogen in astrocytes, but also generate the TCA cycle intermediates succinyl-CoA and acetyl-CoA (41). However, although isoleucine provides some cerebral glutamate nitrogen this contribution is likely modest (41). Nevertheless, an analysis by Rothman et al. (25) suggests that the BCAA/BKCAA shuttle in brain is feasible. But, as pointed out by Somersalo and Calvetti (42) the proposed BCAA/BCKA shuttle is problematic because the GDH reaction is suggested to proceed in the direction of reductive amination of α-ketoglutarate in the neurons and oxidation of glutamate in the astrocytes. As noted above, relatively little label derived from intracarotid [^13^N]ammonia is incorporated into brain glutamate in MSO-treated rats even when GS is inhibited 85% and the animals are hyperammonemic. Under these conditions, compartmentalization of ammonia metabolism in the brain is disrupted such that blood-derived [^13^N]ammonia, which would normally have been efficiently trapped as glutamine (amide) in astrocytes, freely mixes with the neuronal ammonia pool. If the GDH reaction were important for the *net* synthesis of glutamate in neurons considerable label should have been present in brain glutamate in the MSO-treated rats. The fact that this was not observed suggests that the GDH reaction is not important for the net synthesis of glutamate in neurons even under hyperammonemic conditions. Thus, although transfer of leucine and other BCAAs between neurons and astrocytes is feasible and much evidence suggests that leucine is transaminated in astrocytes, the GDH reaction is unlikely to play a major role in any BCAA/BCKA shuttle.

As the above discussion attests, glutamate/glutamine homeostasis in astrocytes is still not fully understood. Somersalo and Calvetti (42) offer a mechanism for balancing nitrogen and carbon metabolism in the brain by suggesting that alanine derived from transamination of pyruvate with glutamate in the neurons (Eq. 4) is taken up by astrocytes, where the reverse reaction transfers the amino group from alanine to α-ketoglutarate (Eq. 5). Concomitantly, α-ketoglutarate is transaminated with leucine in the neurons to generate glutamate and KIC by the action of BCATc. This KIC is taken up by the astrocytes where it is transaminated with glutamate to generate leucine and α-ketoglutarate via a reaction catalyzed by BCATm. [The direction of nitrogen flow is the opposite of that shown in Eqs 6 and 7.] According to their Fig. 10 the combined action of the alanine shuttle and the GDH reaction replenishes the ammonia pool in the astrocytes.

The Somersalo and Calvetti model will be useful for further studies of cerebral nitrogen metabolism. The results of our ^13^N tracer studies suggest that nitrogen shuttles requiring operation of the GDH reaction in opposite directions in astrocytes and neurons are unlikely. In the model suggested by Somersalo and Calvetti the GDH reaction proceeds in the direction of oxidative deamination of glutamate in *both* compartments in agreement with our previous findings (see their Fig 10). The Somersalo and Calvetti model also requires a net movement of ammonia from neurons to astrocytes, consistent with our results obtained with [^13^N]ammonia. A major source of ammonia in the neurons is the PAG reaction. However, there are many additional enzyme-catalyzed metabolic reactions that can generate ammonia in both compartments, including the GDH reaction (10). Tracer studies with blood-derived [^13^N]ammonia clearly show that the two metabolic pools of ammonia in the rat brain do not readily mix except under hyperammonemic conditions in which the GS reaction is strongly inhibited (2). Despite the presence of two kinetically distinct ammonia compartments in the normal brain the ammonia generated in the neuronal compartment must be disposed of eventually. Since the GDH reaction in neurons is unlikely to participate in the net removal of ammonia and the brain does not have a complete urea cycle, the overwhelming route for removal of neuron-derived ammonia is the GS reaction in astrocytes. Evidently, under normoammonemic conditions the GS reaction removes ammonia efficiently from the astrocyte pool at a rate that ensures a net movement/gradient of ammonia from neurons to astrocytes, preventing uniform mixing of ammonia in the small and large compartments. In this regard, previous work has shown that ammonia is passively and actively rapidly transported into astrocytes in culture (43).

An unresolved issue is the nature of the transamination partners required for net conversion of α-ketoglutarate to glutamate in astrocytes. As noted above, neuronal-derived alanine is a possible source (42), but the contribution of alanine may be quantitatively minor under normoammonemic conditions. Another possibility is neuron-derived aspartate (Eqs 8 and 9) as suggested by Pardo et al. (24). In many respects, aspartate is a more attractive transamination partner in astrocytes than is alanine as a source of glutamate nitrogen. Aspartate aminotransferase (ASPAT) is extremely active in most tissues and the components of the reaction are thought to be at or near thermodynamic equilibrium in brain (44, 45) and liver (46). As a result, nitrogen is exchanged extremely rapidly between glutamate and aspartate in these tissues (23, 45, 47). Moreover, ASPAT is present in cytosolic and mitochondrial compartments. Thus, any model of nitrogen flux among amino acids that are transaminated with α-ketoglutarate in the brain must take into account nitrogen exchange between glutamate and aspartate in mitochondria and cytosol. A variant of the model proposed by Pardo et al. (24) has been suggested by Hertz (48). In the Hertz model ASPAT-catalyzed transamination of aspartate with α-ketoglutarate in astrocyte cytosol is retained. However, the “missing” aspartate is generated in the astrocytic mitochondria by transamination of glutamate with oxaloacetate rather than from the mitochondrial ASPAT reaction in neurons as envisaged by Pardo et al. (24).

(8) $$\begin{array}{llll} & \text{L-Glutamate}+\mathrm{oxaloacetate}\to \alpha -\mathrm{ketoglutarate}\; & & \;\\ & \;+\text{L-aspartate (neurons)} \end{array}$$

(9) $$\begin{array}{llll} & \text{L-Aspartate}+\alpha -\mathrm{ketoglutarate}\to \mathrm{oxaloacetate}\; & & \;\\ & \;+\text{L-glutanate (astrocytes)} \end{array}$$

Finally, the importance of linked aminotransferase reactions in the brain should be emphasized. The capacity of the brain to catalyze α-ketoglutarate- and glutamate-linked aminotransferase reactions with a large number of amino acids/α-keto acids is considerable (33). As noted above, the brain has the capacity to take up many amino acids from the circulation, including BCAAs. These amino acids can then contribute to the astrocytic pool of glutamate nitrogen via α-ketoglutarate-linked aminotransferase reactions.

A diagram of the major pathways contributing to cerebral nitrogen homeostasis is shown in Figure 1. Summary: (1) Ammonia for glutamine synthesis in astrocytes is obtained by diffusion from blood and CSF, and from numerous endogenous enzyme-catalyzed reactions, but principally the PAG reaction (in neurons) and the GDH reaction (in both astrocytes and neurons). (2) To maintain nitrogen balance glutamine is exported to blood/CSF. (3) The net GDH-catalyzed conversion of glutamate to α-ketoglutarate and ammonia in astrocytes results in a drain on glutamate in these cells that cannot be replenished by the glutamate–glutamine cycle (or GABA–glutamine cycle). (4) Conversion of α-ketoglutarate back to glutamate in astrocytes can be accomplished by α-ketoglutarate-linked aminotransferases, which are well represented in brain. (5) Transamination partners in the astrocytes include neuron-derived alanine (less important) and aspartate (more important). (6) ASPAT is crucial in maintaining cerebral nitrogen balance. (7) Additional transaminase partners in the astrocytes include amino acids taken up across the BBB, most notably the BCAAs. Evidently, much work remains in elucidating mechanisms contributing to cerebral nitrogen flux/homeostasis.

**Figure 1:**
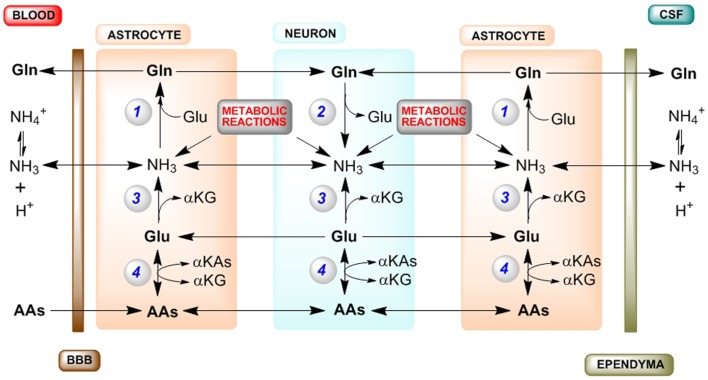
**Major routes for nitrogen homeostasis in the brain with emphasis on the central importance of ammonia**. Abbreviations: AA, amino acid; αKA, α-keto acid; αKG, α-ketoglutarate; A–V, arterial–venous; BBB, blood–brain barrier; CSF, cerebrospinal fluid; GDH, glutamate dehydrogenase; GS, glutamine synthetase; PAG, phosphate-activated glutaminase; PC, pyruvate carboxylase. Enzyme reactions: (1) GS, (2) PAG, (3) GDH, (4) various αKG/glutamate-linked aminotransferases. Tracer studies with [^13^N]ammonia have shown that about 25–40% of the tracer is taken up in a single pass through the brain (2, 5, 6). Most of this blood-derived [^13^N]ammonia (as well as CSF-derived [^13^N]ammonia) is rapidly trapped in the astrocyte compartment as L-[*amide*-^13^N]glutamine by the action of GS. The cerebral trapping of blood-derived [^13^N]ammonia suggests that there should be an A–V difference for ammonia across normal brain. However, the concentration of ammonia in blood is relatively low (<40 μM in normal human blood and <80 μM in normal rat blood). As a result, a cerebral A–V difference for ammonia is difficult to measure in normoammonemia, although it is well documented to occur during hyperammonemia [reviewed by (10)]. A net cerebral uptake of many AAs has been demonstrated for healthy human volunteers in the post absorptive state (9). In order to maintain cerebral nitrogen balance some glutamine is released to the blood/CSF, a process that is more pronounced during hyperammonemia [reviewed by (10)]. As a result, the concentration of glutamine in the normal CSF/extracerebral fluid is relatively high and much higher than that of any other amino acid (49). Many endogenous reactions contribute to the cerebral ammonia metabolic pool, but especially the PAG and GDH reactions (10). As a result of rapid removal of this ammonia as glutamine there is an ammonia concentration gradient from the neurons to the astrocytes, maintained by diffusion of NH_3_ and active transport of ${\text{NH}}_{4}^{+}$ [c.f. (43)]. During the glutamate–glutamine cycle, approximately one equivalent of nitrogen enters the astrocytes as glutamate and approximately two equivalents exit as glutamine. One equivalent of nitrogen in glutamine is readily obtained from ammonia, which is incorporated into the amide position. The other nitrogen in glutamine is derived from glutamate. However, a portion of this glutamate is metabolized to αKG and ammonia by the action of GDH. Moreover, some carbon/nitrogen originating in glutamate is lost when glutamine exits the brain. Glutamate carbon may be replenished in astrocytes by anaplerotic mechanisms, most notably pyruvate carboxylase (PC). [PC activity is present exclusively in astrocytes, and its activity has been estimated to be about 37% that of the cerebral glutamine synthesis rate (50).] Glutamate nitrogen is replenished in the astrocyte by transamination reactions between αKG and a suitable AA, generating the corresponding αKA. Leucine is an especially favorable aminotransferase partner in astrocytes, because (1) it is readily taken up across the BBB (9), (2) blood-derived leucine is metabolized in the small compartment (i.e., astrocytes) (37, 38), and (3) both astrocytes and neurons contain appreciable branched-chain amino acid aminotransferase activity (34, 35). Another possible aminotransferase partner in the astrocytes is aspartate (24, 48). Note: (1) for simplicity, the GABA–glutamine cycle is not shown as it is considerably slower than the glutamate–glutamine cycle; (2) endogenously generated ammonia is depicted as NH_3_ (but represents ${\text{NH}}_{3}+{\text{NH}}_{4}^{+}$); (3) enzyme cofactors are not shown; (4) tracer studies show that the cerebral GDH reaction is reversible, but as discussed in the text the net direction of the GDH reaction in both astrocytes and neurons is likely in the direction of glutamate oxidation to αKG and ammonia; and (5) possible movements of αKAs between neurons and astrocytes (required for carbon balance) are not shown.

## Conflict of Interest Statement

The author declares that the research was conducted in the absence of any commercial or financial relationships that could be construed as a potential conflict of interest.
